# BARRIERS AND FACILITATORS TO PROVIDING COVID-19 CONTACT TRACING INFORMATION AMONG ADULT NIGERIANS

**DOI:** 10.21010/Ajidv19i2S.4

**Published:** 2025-10-17

**Authors:** ODIKPO Linda Chihurumnanya, BALAS Ayuba, UBAH Chinenye, MBADUGHA Chisom, NDUBUISI-OKOROEZI Lovelyn, IKECHUKWU-OKOROEZI Jennifer

**Affiliations:** 1 Department of nursing science, Nnamdi Azikiwe University Awka, Nnewi Campus,Nigeria; Gombe State Hospital Services Management Board, Nigeria; Lecturer,Adult Nursing, Anglia Ruskin University Cambridge; Staff Nurse-older adults Mental health unit, Sheffield health and social care NHS Foundation trust; Clinical Nurse Educator, East Kent Hospital University, NHS Foundation trust; Senior Eating Disorder Practitioner,NAVIGO Health and Social CIC England, United Kingdom; 2 Department of nursing sciences, Gombe State University, Nigeria; 3 Anglia Ruskin University, United Kingdom; 4 Head of Clinical Education, United Lincolnshire Teaching Hospital NHS Trust

**Keywords:** Barriers, Facilitators, COVID-19, Contact tracing, Information, Nigerians

## Abstract

**Background –:**

Contact tracing information as one of the measures of COVID-19 control had been met with some barriers and facilitators. This study was set to identify barriers and facilitators to providing COVID-19 contact tracing information among adult Nigerians.

**Materials and Methods –:**

The study was a prospective web-based cross-sectional descriptive design. Multi-stage sampling technique was used to select 1015 adult Nigerians within 18-70 years age band. Data were collected through Google forms and analyzed with the aids of Statistical Package for Social Sciences version 22.

**Results –:**

The majority (91.1%) of the respondents indicated that they will provide contact tracing information if they were confirmed or suspected to have COVID-19. The most identified facilitator to providing Covid-19 contact tracing information by the majority (90.7%) of the participants was to help stop the spread of COVID-19. More than average (58.9%) of participants did not trust the authorities; this was the most identified barrier to providing COVID-19 contact tracing information. The intent to provide contact tracing information was significantly associated with being a healthcare professional (p = .007) and place of residence (p = .044).

**Conclusion –:**

This study has identified facilitators and barriers to providing COVID-19 contact information. Despite the progress in management of COVID-19, the future is not predictive; therefore, providing contact tracing information will remain relevant in planning strategic and specific interventions for controlling infectious diseases. Government should make more effort to improve public trust on confidentiality of information and governance in general.

## Introduction

Coronavirus disease 2019 (COVID-19) caused by the SARS-CoV-2 virus was a pandemic which represented a major global public health disaster (Odette *et al.*, 2020). Testing, contact tracing and isolation strategies including vaccination have been seen as the most effective methods of controlling the COVID-19 outbreak (Baraniuk 2020; Keeling *et al.*, 2020; Kucharski, 2020; Odikpo *et al.*, 2022).

Contact tracing and case isolation are common methods for controlling infectious disease outbreaks. However, the effectiveness of any contact tracing system rests on public engagement (Odette *et al.*, 2020). It is one critical area that requires specific attention, which is engagement with the contact tracing element of the system. Different countries including Nigeria approached contact tracing in different ways, but the core elements are the same, which aimed at preventing onward transmission of an infectious disease. This can be achieved by identifying, assessing and managing people who have been in close contact with an infected individual through the obtained accessible COVID-19 related information which has proven to be beneficial in this regard (Ndubuisi-Okoroezi *et al.*, 2022; Ubah *et al.*, 2022). Contact tracing as was done during COVID-19 in Nigeria may not be unique to the COVID-19 pandemic as it has been used extensively in previous emerging infectious disease outbreaks (Saurabh and Prateek, 2017).

Despite the fact that contact tracing with other protocols is an effective way for COVID-19 infection containment, certain barriers influence contact tracing on one end while others facilitate it at the other end. Some of these barriers have been reviewed by different authors in similar studies including studies by Jansen-Kosterink *et al*. (2020); Thomas *et al*. (2020) and Williams *et al*. (2020). The authors identified privacy concerns as one the barriers to COVID-19 contact tracing especially over government surveillance and desire to protect private life as contact tracing was seen as an intrusion into the domain of private life and living (Caleo *et al.*, 2018). Other identified barriers were mistrust and apprehension (Greiner *et al.*, 2015; Bachtiger *et al.*, 2020), unmet need for more information and fear of the negative implication it may have on their economic life and fear of stigmatization**.** El-Sadr *et al*. (2022) identified stigma as a major barrier to effective tracing efforts. Some of the facilitating compliance factors to contact tracing were desires to reduce risk to oneself and one’s family and friends and to, a lesser extent, the general public (Wright *et al.*, 2022). Also of importance were a desire to return to normalcy, the availability of activities and technological means to contact family and friends, and the ability to work from home (Wright *et al.*, 2022). The**se** factors which may be likened to barriers that may influence an individual’s willingness to engage with a contact tracing system, hence an understanding of these factors have become urgent, considering the unpredictable nature of infectious disease outbreak like COVID-19 pandemic. This is necessary in order to put into work, best practices that may facilitate contact tracing (Nachega *et al.*, 2021). This is especially so in Nigeria where everything concerning COVID-19 is surrounded by a lot of myths and misinformation. It was on these premises that the authors decided to embark on a national study to identify the barriers and facilitators to COVID-19 contact tracing in Nigeria as contact tracing is important in controlling community transmission of COVID-19 pandemic and other infectious diseases. This study assessed the barriers to providing COVID 19 contacts information among adult Nigerians within 18-70years of age. It determined facilitators to providing COVID 19 contacts information among adult Nigerians between 18-70years and also examined the association between provision of contact information and socio-demographic variables.

## Materials and Methods

**Study design, setting and participants -** This study was a prospective web-based cross-sectional descriptive design conducted nationwide in Nigeria. The participants were adult Nigerians between 18-70 years of age from the North, South, East and West regions of Nigeria.

**Sample and sampling technique–**The study was a national survey where 1015 respondents participated in the study at a 95% level of confidence. Due to the heterogeneity of the population, multi-stage sampling technique was used. Stage one involved purposive selection of the North, South, East and West regions of Nigeria. The second stage involved the selection of all six geopolitical zones of Nigeria plus Abuja (the Federal Capital Territory), followed by selection of two States with the highest number of COVID-19 cases from each of the six geopolitical zones. These States were Kano and Kaduna from the North West; Gombe and Bauchi from the North East; Kwara and Plateau from the North Central; Lagos and Oyo from the South West; Enugu and Ebonyi from the South East; Rivers and Delta from the South-South; and Abuja. The third stage involved stratified division of selected towns in each selected zone into rural and urban locations where a simple random sampling was used to select two towns each from the urban and rural categories. Finally, the eligible participants accessed the survey online where the initial respondents were encouraged to assist the researchers to recruit participants from their acquaintances in the chosen towns.

As a web-based study, the final random selection of the eligible participants was not readily feasible. The researchers have limited control on the measure to get a fair representation of various socio-economic groups and access to internet/digital literacy. These factors were regarded by the researchers as some of the limitations of the study to mitigate biases that may influence the outcome of the study.

**Instrument for data collection -** Data were collected using an adapted survey instrument developed by the World Health Organization (WHO, 2020) to guide states that wish to conduct behavioral insight studies related to COVID-19. Variables adapted from the WHO’s instrument include socio-demographic, COVID-19 experience, trust in institutions, and testing and tracing. However, under tasting and tracing, only tracing aspect was adapted to identify barriers and facilitators (drivers) to contact tracing. The instrument comprised sections A, B and C. Section A contained questions on the socio-demographic characteristics of respondents where age, sex, level of education, health professional and rural/urban location were included, while chronic illness, household and financial situation were excluded. Section B contained questions on facilitators to providing COVID-19 contact tracing information, while section C contained questions to determine barriers to providing COVID-19 contact tracing information. To fit into the Nigerian context for the study, the survey tool was validated by a group of experts well-grounded in behavioral researches and for developing and validating survey instrument similar to this tool. These experts included a public health expert, an epidemiologist and two clinical researchers who determined the adequacy and precision of the survey items. The final draft of the validated questionnaire was pilot-tested in Nigeria with a sample of 100 Nigerians with same characteristics as the study population who were not part of the study. This was to ensure clarity of survey items before broad use.

The score obtained was subjected to reliability test using Cronbach’s Alpha and a coefficient of 0.8 was obtained which was considered strong.

**Ethical approval -** Ethical approval was obtained from the National Human Research Ethics Committee of the University of Nigeria Teaching Hospital, Ituku-Ozalla Enugu State with Reference No: (NHREC/05/01-2008B-FWAOOOO2458-1RB00002323). Data were obtained anonymously with the consent of the participants, participants were also informed about the confidentiality of all information they would provide during and after the study, and that the information will be used only for the study.

**Method of data collection -** Data were collected online through Google Forms. The researchers and six research assistants from each selected geopolitical zone disseminated the survey instrument online via Whats App and emails. Initial respondents were requested to share further with their contacts that are residents or indigenes of the selected zones. The questionnaire was launched online nationwide on the same date.

**Method of data analysis -** Data were analyzed with the aids of the Statistical Package for Social Sciences (SPSS) version 22. Descriptive statistics was applied in the analysis of frequencies and percentages to summarize the items in the questionnaire. The inferential statistics was used for the Chi-Square Test of Independence to ascertain the relationship between the intent to provide contact information and the socio-demographic characteristics of the participants at a 5% level of significance.

## Results

**Table 1:** Demographic Characteristics of the Respondents n = 1015

	Frequency	Percent	Range	M±SD
Age			18-70	29.84±9.11
-< 30	597	58.8		
-30-39	282	27.8		
-40-49	80	7.9		
-50+	56	5.5		
Sex				
-Male	376	37.0		
-Female	639	63.0		
Educational qualification				
-Primary	10	1.0		
-Secondary	82	8.1		
-Tertiary	923	90.9		
Healthcare professional				
-Yes	521	51.3		
-No	494	48.7		
State of origin				
-North	191	18.8		
-South	142	14.0		
-East	622	61.3		
-West	60	5.9		
City of residence				
-North	250	24.6		
-South	189	18.6		
-East	446	43.9		
-West	130	12.8		
Location				
-Urban	863	85.0		
-Rural	152	15.0		

Table 1 shows the demographic characteristics of the respondents. Their age ranged from 18-70 years with mean and standard deviation, 29.84±9.11 and modal age group, below 30 years (58.8%) years. Females (63.0%) were more than males (37.0%); almost all had tertiary education (90.9%) and about average were healthcare professionals (51.3%). Majority of them were from the East (61.3%) and also resided in the East (43.9%); most were urban residents (85.0%).

**Table 2:** Contact with Confirmed COVID-19 Patient n = 1015

	Frequency	Percent
Had contact with a confirmed COVID-19 patient		
-Yes	87	8.6
-No	654	64.4
-Don’t know	274	27.0

From Table 2, findings show that only very few had contact with confirmed COVID-19 patient (8.6%); majority never had any contact (64.1%) while 27.0% were uncertain.

**Table 3:** Facilitators to Providing COVID-19 Contacts Information n = 1015

	Frequency	Percent
What if you tested positive, and was asked by NCDC to share the names of people you have been in contact with, will you provide their names?		
-Yes	925	91.1
-No	90	8.9
If yes, what are your reasons? *(n = 925)*		
-It will help to stop spread of COVID-19	839	90.7
-It is my responsibility as a citizen	409	44.2
-Will face penalties if I don’t	40	4.3
-To protect other people from being infected	748	80.9
-My families and friends will expect me to do that	178	19.2
-To notify those contacts to enable them take appropriate actions	623	67.4

Findings in Table 3 show that if they tested positive, almost all were willing to provide the names of people they had contact with to NCDC (91.3%). The major facilitators to this included: to help stop spread of COVID-19 (90.7%), to protect other people from being infected (80.9%) and to notify those contacts to enable them take appropriate actions (67.4%).

**Table 4:** Barriers to Providing COVID 19 Contact Information n = 90

	Frequency	Percent
If no, what are your reasons? *(n = 90)*		
-I don’t trust the authorities	53	58.9
-I won’t want others to know I tested positive	11	12.2
-My family will blame me for providing their names	23	25.6
-My families and friends will expect me not to share their names	18	20.0
-I could contact them myself	20	22.2
-It could lead to loss of income for those people I gave out their name due to quarantine or isolation	15	16.7
-Those people would blame me for sharing information that led to their loss	17	18.9

From Table 4, the major barrier to providing COVID-19 contact information if test is positive was lack of trust for authorities (58.9%). Being blamed by people for providing their names (25.6%), contacting the people by oneself (22.2%) and families and friends expecting not to share their names (20.0%) were also barriers though among fewer persons.

**Table 5:** Relationship between Intent to Provide Contact Information and Socio-demographic Characteristics of the Respondents

	Intent to Provide Contact Info			Chi-square	p-value
	Yes	No	Total		
Age				7.361	.061
-< 29	532(89.1)	65(10.9)	597		
-30-39	265(94.0)	17(6.0)	282		
-40-49	75(93.8)	5(6.3)	80		
-50+	53(94.6)	3(5.4)	56		
Sex				1.491	.222
-Male	348(92.6)	28(7.4)	376		
-Female	577(90.3)	62(9.7)	639		
Educational level				3.468	.063
-Primary & Secondary	79(85.9)	13(14.1)	92		
-Tertiary	846(91.7)	77(8.3)	923		
Health care professional				7.260	.007
-Yes	487(93.5)	34(6.5)	521		
-No	438(88.7)	56(11.3)	494		
State of origin				2.636	.451
-North	177(92.7)	14(7.3)	191		
-South	133(93.7)	9(6.3)	142		
-East	560(90.0)	62(10.0)	622		
-West	55(91.7)	5(8.3)	60		
State residence				5.824	.121
-North	237(94.8)	13(5.2)	250		
-South	171(90.5)	18(9.5)	189		
-East	399(89.5)	47(10.5)	446		
-West	118(90.8)	12(9.2)	130		
Location				4.073	.044
-Rural	793(91.9)	70(8.1)	863		
-Urban	132(86.8)	20(13.2)	152		

From Table 5 results, intent to provide contact information was significantly related with being a healthcare professional (p = .007) and location of residence (p = .044). The intent was significantly higher among healthcare professionals (93.5%) than non-healthcare professionals (88.7%). For location, the rural residents (91.9%) were associated more with the intent than the urban residents (86.8%).

## Discussions

Among the facilitators of contact sharing in contact tracing is the eagerness to curtail the spread of the Covid scourge. Contact sharing was also facilitated by a sense of duty to protect others. The understanding that control of spread of disease is a shared responsibility is recognized by the World Health Organisation (2016) as a long-lasting public health strategy. This opinion is also echoed in the findings of Megnin-Viggars *et al*. (2020) where engagement in contact tracing was facilitated by its view as collective responsibility by the people and personal benefits of curtailing the spread. Since this understanding is widespread, one would expect a high compliance with contact tracing. However, contact tracing is disappointingly low due to correctable factors. Thus, creating a paradox between the participants’ intentions to protect the community and the practicalities of achieving it. Contact tracing has been modeled as a crucial strategy in curtailing the spread of covid-19 (Giordano *et al.*, 2020; Kucharski *et al.*, 2020). Even though the review by Juneau *et al*. (2023) showed that the certainty of controlling spread of Covid-19 spread through contact tracing was low but increased when it was combined with other interventions such as self-isolation. Hossain *et al*. (2022) argued that the effectiveness of contact tracing in preventing spread could not be captured due to other complimentary interventions. Perhaps, outside security concerns, participants’ doubt in the potential of contact tracing in reducing spread may have affected compliance. But it is beyond the scope of this study to understand the perception about contact tracing.

Based on evidence from the study, the major reason participants withheld their contact details is related to mistrust in the authorities. People’s opinions were strongly reflected in these statements: ‘My families and friends will expect me not to share their names’ or’ Those people would blame me for sharing their name’. This clearly shows lack of confidence in the way their information may be handled when shared. The overarching barrier to contact tracing in Nigeria remains an unwillingness to share personal information with the government even on covid-19 matters. In a similar study, Megnin-Viggars *et al*. (2020) reported that privacy concerns; mistrust and/or apprehension around supplying contact information hindered contact tracing during disease outbreaks and deterred sharing of contact information. In a rapidly growing cosmopolitan world, information management is crucial to people’s life and security. It is therefore expected that every sector should exercise more vigilance on information matters. Agreeably, a lot could be obtained with name, address, and phone details if security measures are not used to protect such information. This leaves the nation with a short-term option of exploring other alternatives to control the spread of infection and a long-term option of restoring the people’s faith in governance.

The benefits of contact sharing are not limited to controlling Covid-19 pandemic. In the long run, it will facilitate an understanding of the impact of information sharing on other health conditions. In essence, to build the trust of the populace in the government’s ability to handle contact information, there should be a reform in information management. As the nation recovers from the impact of Covid-19 pandemic, management of possible future disease outbreaks through information sharing will rely on tactful information governance. It is important to note that if people perceive sharing their contact as a threat to their security, the barriers for them will outweigh the benefits (Syed-Abdul *et al.*, 2016) and makes it unlikely that they will share their details.

Interestingly, there was a significant difference between participants’ intention to share contact and their profession and location. It was found that health care professionals are more willing to share their contact to prevent spread than non-healthcare professionals. This observation could be explained by the health belief model where cues such as knowledge of Covid-19 can spur people to participate in health promotion. (Syed-Abdul *et al.*, 2016). It implies that the more health information people have, the more likely they are to engage in preventive practices because they understand why it is necessary. Intention to share contact details also differed significantly with participants’ location (rural/urban) with rural dwellers showing more willingness to share information than urban dwellers. Given that the impact of Covid-19 was higher in urban cities in terms of morbidity and mortality rate (Dixon, 2020), the result calls for a more reliable intervention to prevent spread irrespective of location. The authors considered that participants’ perception of the process of information management may differ between urban and rural dwellers but again it is beyond the scope of this study to understand why the difference exists.

### Implication

The nexus of this study is to reflect and learn from the nation’s recent response to the Covid-19 pandemic and be preemptive in case of future emergencies. While the eradication of Covid-19 is widely targeted, the future is not predictive of disease outbreaks. Alongside, misinformation and stigma, and poorly sustained adherence to isolation, Nachega *et al*. (2021) identified contact tracing as part of the challenges facing African countries. Therefore, this result will be useful in critically evaluating the country’s response to Covid-19 and conditions that inhibited success, for improvement and future intervention plans. In addition, addressing people’s unwillingness to share contact due to fear of how it will be managed requires strengthening of information governance by the government. There is need to implement measures that ensure transparency in how information is collected and managed safely. Enactment of laws that protects private information will promote compliance with information sharing. Another important strategy identified in Nachega, *et al*. (2021) as a good practice is strengthening wider community engagements to promote awareness and compliance with preventative measures.

## Conclusion

The study examined the barriers and facilitators to contact tracing in Nigeria using a descriptive survey approach. It was found that people recognize contact tracing as a shared responsibility between citizens and the government, and would share their contact based on the need to control spread of covid-19. However, they did not engage or comply with contact tracing because they do not trust the process. This widespread mistrust issue calls for more tact in information management to restore the trust of the populace in the prevention strategies. It also raises an ethical question on whether the government has a duty to protect the information shared by its citizens and whether they are willing to take responsibility should their confidentiality be breached. It would also be worth exploring, other sustainable approaches to contact tracing to improve compliance. The results also showed that intentions to comply with contact tracing are significantly higher among health care workers and rural dwellers than people in other professions. Evidence in how people responded to contact tracing is scanty, making this an outstanding one in Nigeria. Information here should be used to prepare the nation for future emergencies.

**Conflict of interest**: The authors declare that there is no conflict of interest associated with this study.

**Funding:** The study was self-funded by the authors as no external fund was received for the study.

**List of Abbreviations:**
COVID-19: Coronavirus disease 2019;
WHO: World Health Organization;
NCDC: Nigeria Centre for Disease Control.
